# Acute or Chronic β‐Caryophyllene Systemic Administration in Healthy Adult Male Mice Does Not Modulate Anxiety‐Like Extinction Behavior Induced by Subsequent Re‐Exposure to 3D Maze

**DOI:** 10.1111/fcp.70049

**Published:** 2025-09-26

**Authors:** Rayan Fidel Martins Monteiro, Marcos Vinícius Lebrego Nascimento, Klinsmann Thiago Lima, Valdina Solimar Lopes Cardoso, José Ramon Gama Almeida, Wellington Junior Taisho Nagahama Costa, Anderson Valente‐Amaral, Bruno Eduardo Godinho Teixeira, Ludmila Santos‐Barbosa, Vinicius Teles Shirakura, Gilmara de Nazareth Tavares Bastos

**Affiliations:** ^1^ Laboratory of Neuroinflammation, Institute of Biological Sciences Federal University of Pará Belém Pará Brazil; ^2^ Laboratory of Reproductive and Development Biology Federal University of São Paulo São Paulo Brazil

**Keywords:** anxiety disorders, anxiety‐like behavior, anxiety‐like‐extinction behavior, cannabinoid type II receptor, endocannabinoid system, β‐caryophyllene

## Abstract

Pharmacotherapy for major anxiety disorders continues to present dangerous side effects, complicating precise treatment choices for each patient. In this context, β‐caryophyllene (BCP), a selective agonist of the cannabinoid type II receptor (CB2R), is recognized as a safe immunomodulatory drug. CB2R has recently been identified in glutamatergic and dopaminergic neurons, supporting its potential as a pharmacotherapy for mood disorders. Thus, we propose to investigate the effects of systemic BCP treatment (50, 100, and 200 mg/kg) on anxiety‐like behavior. To this end, we utilized 92 adult male Swiss mice across two acute and one chronic pharmacological assay using the 3D maze test. In the acute assay, 30 min after treatment (BCP or vehicle), we conducted the One‐Trial Protocol (OTP) lasting 12 min and the Two‐Trial Protocol (TTP) lasting 12 min (comprising two trials of 5 min, with a 2‐min interval between them). In the chronic assay, after 10 days of treatment (once daily; BCP or vehicle), testing was performed over five consecutive days (once daily; 12 min), 30 min after administration of BCP or vehicle. Additionally, locomotion was assessed. Under these conditions, we observed no effects on locomotion, anxiety‐like behavior, or anxiety‐like extinction behavior following either acute or chronic oral administration of BCP. Furthermore, we propose the use of TTP in the 3D maze as a valuable method for assessing acute pharmacological effects in mice. Lastly, behavioral modulation induced by CB2R agonists, particularly BCP, must be further investigated to better understand its potential neurological treatment applications and associated side effects.

## 1 Introduction

The pharmacotherapy for the main anxiety disorders (generalized anxiety disorder, panic disorder/agoraphobia, social anxiety disorder) is based on selective serotonin reuptake inhibitors (SSRIs) and selective serotonin‐norepinephrine reuptake inhibitors (SNRIs) due to better efficacy and less risk against other pharmacological treatments for anxiety disorders such as benzodiazepines [1]. In addition, pharmacotherapy is more effective than psychotherapy [2]. However, SSRIs and SNRIs show dangerous side effects that could discontinue the treatment in many patients. Thus, there is a demand for new, safer pharmacotherapy for anxiety disorders.

In this scope, β‐caryophyllene (BCP) is a known as safe immunomodulatory drug [3, 4, 5, 6, 7]. In addition, BCP was approved by USFDA (United States Food and Drug Administration) as a “food additives permitted for direct addition to food for human consumption” (no. 21CFR172.515). In summary, BCP is a cannabinoid type II receptor (CB2R) selective agonist [8]. CB2R as a target for neurological conditions have been researched as an alternative to psychoactive effects of cannabinoid type I receptor (CB1R) agonists [9], or anxiogenic‐like CB1R effects [10]. As well as BCP is a huge safer drug for consumption for acute or chronic treatment (2000 mg/kg) does not induce toxicity outcomes in mice [11].

Recently, CB2Rs was found by in situ hybridization to be present in glutamatergic and dopaminergic neurons [12, 13, 14]. This finding supports the behavioral effects that are CB2R‐dependent in mice [15]. In addition, it also supports the potential pharmacotherapy of BCP against mood disorders. By this means, some studies report anxiolytic‐like and anti‐depressant‐like effects after BCP systemic and acute treatment in mice tested in current mazes of mood disorders: Bahi et al. (2014) demonstrated these effects 30 min after pre‐treatment with 50 mg/kg of BCP, which were significantly abbreviated by pre‐administration of the CB2R antagonist (AM630) in male C57BL/6 mice [16]; Galdino et al. (2012) demonstrated anxiolytic‐like effects 6 min after pre‐treatment with 50, 100, and 200 mg/kg of BCP in male Swiss mice [17]; and more recently, Oliveira et al. (2020) demonstrated anxiolytic‐like and anti‐depressant‐like effects after pre‐treatment with 100 and 200 mg/kg of BCP in female Swiss mice [18].

Interestingly, as described above, the behavioral BCP effects (e.g., anxiolytic‐like and anti‐depressant‐like) were evaluated in acute experiments. These facts were supported by previous works, which suggest that BCP had these effects [13, 14, 15, 16]. The behavioral BCP effects after chronic administration are yet to be evaluated, as well as anxiety‐like extinction behavior (ALEB) over acute or chronic challenges. The most unconditioned anxiety test must be performed just once [19, 20], which prevents the ALEB from being assessed. In contrast, the 3D maze could be performed over subsequent sessions [21, 22, 23, 24, 25, 26, 27]. That maze consists of a central circular platform connected to 8 arms (distal segment) by 8 bridges (proximal segment), which the arms could be at the same or at a different height from the central platform [24]. As well as in the 3D maze, the bridges and arms segments are delineated without safe places. That model induced the animals to take a risk and not venture far away from the central platform. This avoidance of the distal segment is used as an indicator of fear and anxiety in mice. Thus, when exposed to an unfamiliar radial arm maze (3D maze), mice frequently enter into the proximal segment of an arm of the maze and do not continue into the distal segment initially; over time and over sessions, the distal segments start to be explored. That anxiolytic‐like behavior over time could be interpreted as an ALEB. Thus, ALEB is a kind of anxiety‐like behavior. Hence, in this work, we performed two acute and one chronic assay to assess the behavioral effects of BCP administration in a novelty unconditioned anxiety test: 3D maze. In addition, we evaluated locomotion.

## 2 Methods

### 2.1 Animals and Treatments

The experiments were conducted using 92 male Swiss mice (8–10 weeks old; 20–40 g) housed randomly 4 animals/cage (floor: 451 cm^2^), with sawdust and 1 cardboard tunnel by cage, at 22 ± 2 °C under a 12/12 h light/dark cycle, with access to food and water ad libitum. Mice were obtained from Evandro Chagas Institute (Belém, Pará, Brazil). All animal procedures described in this work were reviewed and approved by the animal ethics committee from the Federal University of Pará (CEUA‐UFPA N° 2 013 280 422). All assays were performed at least 1 week after the animal arrived in the laboratory. All animals were treated with olive oil (vehicle) or BCP (Sigma‐Aldrich/*W225207*). All animals were randomly separated into 4 groups: vehicle, which were treated (5 mL/kg) p.o. (gavage) with olive oil; BCP50, which were treated (5 mL/kg) p.o. (gavage) with 50 mg/kg of BCP diluted in olive oil; BCP100, which were treated (5 mL/kg) p.o. (gavage) with 100 mg/kg of BCP diluted in olive oil; and BCP200, which were treated (5 mL/kg) p.o. (gavage) with 200 mg/kg of BCP diluted in olive oil. For acute assays, the animals were treated once and identified individually with painting on their tails; for chronic assay, the animals were treated 19 times (once a day) and identified individually and randomly with animal ear punch. All cages had one animal from each group (Vehicle, BCP50, BCP100 and BCP200). All animals' procedures were performed in the light phase of the cycle. At the end of the assays, all animals were euthanized with a lethal dose of xylazine and ketamine solution.

### 2.2 Acute Pharmacological Assays

#### 2.2.1 Locomotion Test

The animal's locomotion was assessed to increase the precision of possible BCP effects on anxiety‐like behavior (e.g., increasing in locomotion could be interpretated as an anxiolytic‐like behavior). For the acute assay, 24 animals were required to perform a locomotion test (6 animals/group). All animals were habituated in a behavioral room in a group for at least 1 h without food. After that, the animals were treated (vehicle or BCP), and then, 20 min after the treatment, all animals were individually isolated in another cage (floor: 451 cm^2^) with sawdust. After 10 min, all the animals were tracked by Any‐maze software (*Stoeling Co*) for 20 min using a digital camera. The average speed was calculated by distance traveled divided by mobile time. The test was performed with 80 lx ambient. The animals which spent all time immobile were excluded (vehicle: 1; BCP50: 2; BCP200: 1).

#### 2.2.2 3D Maze Test

The 3D maze (8 arms radial maze variation) apparatus consists of an adaptation in “raised arms” configuration for assessing anxiety‐like behavior [24]. The maze used here consists of an MDF apparatus, with a central octagonal platform measuring 11.2 cm on each side at a height of 60 cm from the floor, eight rectangular “bridges” measuring 15.2 cm × 11.2 cm, and eight rectangular “arms” measuring 35 cm × 11.2 cm. The bridges form an angle of 140° in relation to the surface of the central platform and at the beginning of each bridge there is a small square wall (5 cm × 5 cm) to prevent passage from one bridge to the other. At this configuration, the arms are higher than the central platform. Furthermore, at the end of each arm there is a spatial identification plate (20.2 cm x 11.2 cm).

##### 2.2.2.1 One Trial Protocol (OTP)

OTP was performed to assess anxiety‐like behavior in the BCP acute assay; 24 animals were required to perform the 3D maze test (6 animals/group). There was no previous habituation in the behavioral room to induce a high anxiety spectrum. One hour before treatments, the animals had no access to food; after that, the animals were treated with vehicle or BCP. Thirty minutes after the treatment, the animals were moved gently to the 3D maze. The 3D maze is an adaptation of the raised arm configuration of the original maze presented by Ennaceur et al. (2006) for assessing anxiety in an open space. Our adaptation follows the same measurements; it is made of MDF wood and lined with black contact paper. All animals started the test on the central platform and were tracked by Any‐maze software (*Stoeling Co*) for 12 min using a digital camera. The average speed was calculated by distance traveled divided by mobile time, and an entry was considered when the animal crossed 80% of its body in a zone for at least 1 s. Between each test, the maze was cleaned with alcohol 70%, dry air, and tissue paper. The 3D maze test was performed cage by cage, in which each entry in the maze was randomized by group. The test was performed with 70 lx ambient. Two animals were excluded from the analysis because they spent all tests immobile (BCP50: 1; BCP100: 1).

##### 2.2.2.2 Two‐Trial Protocol (TTP)

TTP was performed to assess ALEB in the BCP acute assay; 24 animals were required to perform the 3D maze test (6 animals/group). All animals were habituated in the behavioral room in the group for at least 1 h without food to induce a low anxiety spectrum. After that, the animals were treated with vehicle or BCP. Thirty minutes after the treatment, the animals were moved gently to the 3D maze. All animals started the test on the central platform and were tracked by Any‐maze software (*Stoeling Co*) twice for 5 min, with a 2‐min interval between the two trials using a digital camera. The average speed was calculated by distance traveled divided by mobile time, and an entry was considered when the animal crossed 80% of its body in a zone for at least 1 s. Between each animal, the maze was cleaned with alcohol 70%, dry air, and tissue paper. The 3D maze test was performed cage by cage, in which each entry in the maze was randomized by group. The test was performed with 70 lx ambient.

### 2.3 Chronic Pharmacological Assay

Chronic pharmacological assay was performed to assess ALEB and locomotion in BCP systemic administration. Twenty animals were required to perform the 3D maze and locomotion test (5 animals/group). The animals started the treatment (vehicle or BCP) at least 1 week after their arrival in the laboratory. The treatment lasted for 18 days (once a day) in this order: 10 days with only treatment; 5 days with treatment and the 3D maze; 2 days with only treatments; and 1 day of locomotion test. One hour before treatments, the animals had no access to food.

#### 2.3.1 3D Maze Test

From 11th to the 15th day of treatment, all animals were habituated in the behavioral room in groups for at least 1 h without food. After that, the animals were treated (vehicle or BCP). Thirty minutes after the treatment, the animals were moved to the 3D maze. All animals started the test on the central platform and were tracked by Any‐maze software (*Stoeling Co*) for 12 min using a digital camera. The average speed was calculated by distance traveled divided by mobile time, and an entry was considered when the animal crossed 80% of its body into a zone for at least 1 s. Between each animal, the maze was cleaned with 70% alcohol, dry air, and tissue paper. The 3D maze test was performed, cage by cage, in which each entry in the maze was randomized by group. The test was performed with 70 lx ambient light for the 5 days of the test. On the 16th and 17th days of the experiment, the animals were treated as in the first 10 days of treatment. One animal was excluded from the analysis because it spent the entire test running (BCP100: 1). In each session, for latency of the first arm entry, the value of 720 s has been assigned for an animal that did not enter the arms; for average time of arm visit, the value of 0 s has been assigned for an animal that did not enter the arms; for arms/bridges ratio entry, the value of 0 has been assigned for an animal that did not enter the arms; for arms/bridges ratio time, the value of 0 has been assigned for an animal that did not enter the arms; for dispersion of exploration, the optical density was performed using Fiji ImageJ software (version 1.54, National Institutes of Health, United States) of heat map with maximum contrast of 7 s (available by Any‐maze software) in gray scale.

#### 2.3.2 Locomotion Test

In the 18th day of experiment, all cages were transferred to behavioral room 1 hour before the test to decrease anxiety to the environment test. At this time, all animals had no access to food. After that, the animals were treated (vehicle or BCP); 20 min after the treatment, all animals were individually isolated in another cage (floor: 451 cm^2^) with sawdust. Ten minutes after, all animals were tracked by Any‐maze software (*Stoeling Co*) for 10 min using a digital camera. The average speed was calculated by locomotion divided by mobile time. The test was performed with 80 lx ambient. One animal was excluded from the analysis because it spent the entire test running (BCP100: 1).

### 2.4 Statistical Analysis

For behavioral tests in bar graphics, one‐way ANOVA was performed followed by Tukey post‐test, where the data were expressed as mean ± SEM. For behavioral tests in line graphics, two‐way ANOVA was performed followed by Tukey post‐test, where the data were expressed as mean ± SEM or mean ± SD. The significant degree considered was *p* ≤ 0.05. All statistical tests were performed in GraphPad Prism software (version 8.0.1).

## 3 Results

### 3.1 BCP Acute Treatment Does not Promote Locomotion Alterations in Swiss Male Mice

**FIGURE 1:**
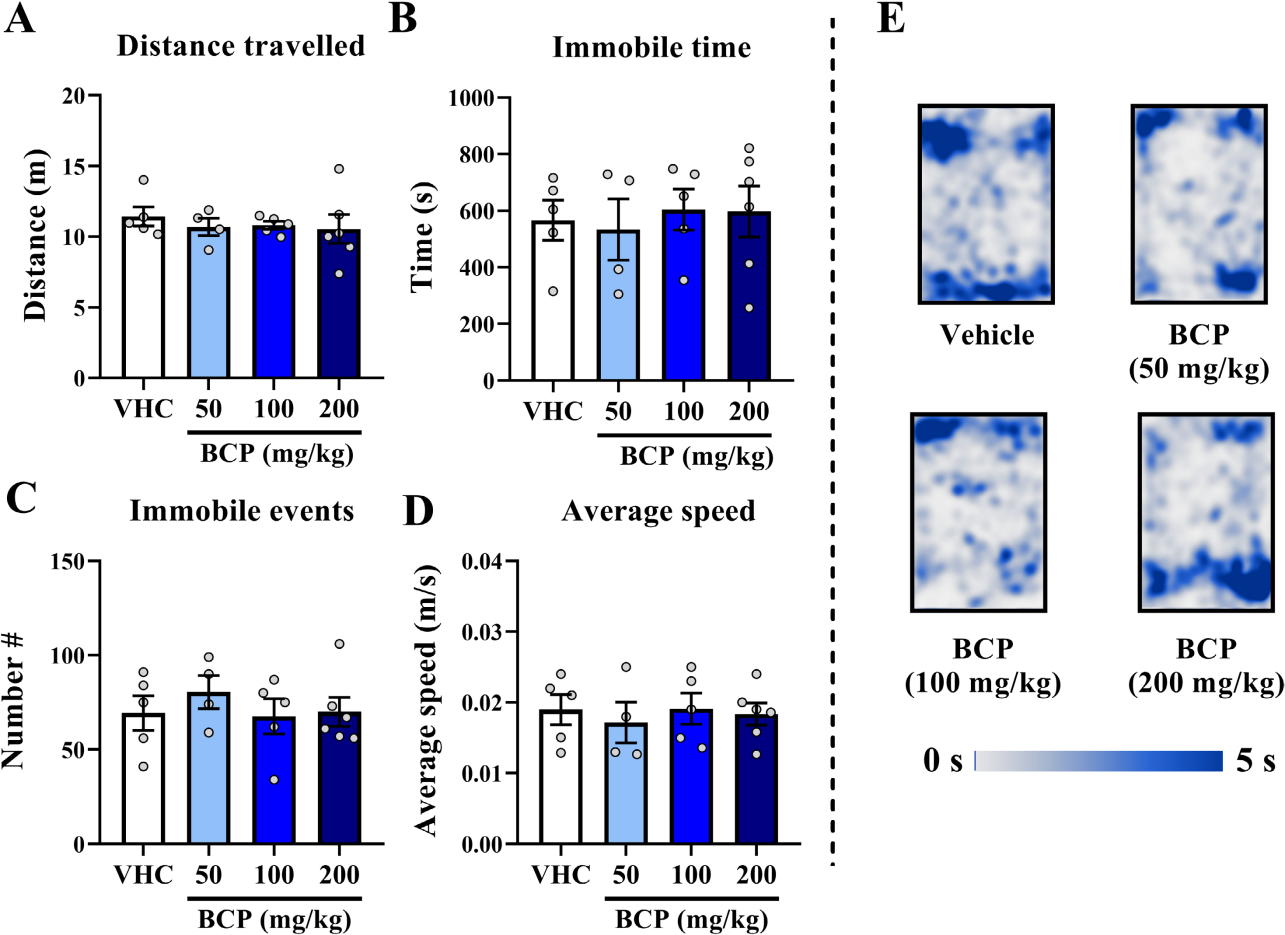
Locomotion results from acute pharmacological assay: The locomotion test was performed 20 min after VHC or BCP oral administrations and the test lasted 20 min, as well as the entries schedule were randomly between groups. (A) Distance travelled (m). (B) Immobile time (s). (C) Number of immobile events. (D) Average speed (m/s): calculated by distance travelled divided by mobile time. (E) Heat map of groups' trajectory. All data were expressed as mean ± SEM At all analyses, there are no significant differences between groups: *p* > 0.05.

In the locomotion test, the distance traveled, time immobile, number of immobile episodes, or average speed was not changed 20 min after acute treatment with a high dose of BCP (50, 100, 200 mg/kg) in comparison to the Vehicle group along with 20 min of test: *p* > 0.05 at all parameters (Figure 1); or along 4 segments of 5 min of test: *p* > 0.05 for distance traveled and time immobile in each segment of test (Figure S1).

In addition, locomotion parameters were analyzed at all acute assays in the 3D maze test (distance traveled, immobile time, number of immobile episodes, and average speed) for all tested times or by 4 segments of 3 min (distance traveled and immobile time) were also not significantly different between groups: *p* > 0.05 at all analyses (Figure S2).

### 3.2 BCP Acute Treatment Does not Promote Anxiety‐Like Behavior or ALEB Alterations in Swiss Male Mice

**FIGURE 2:**
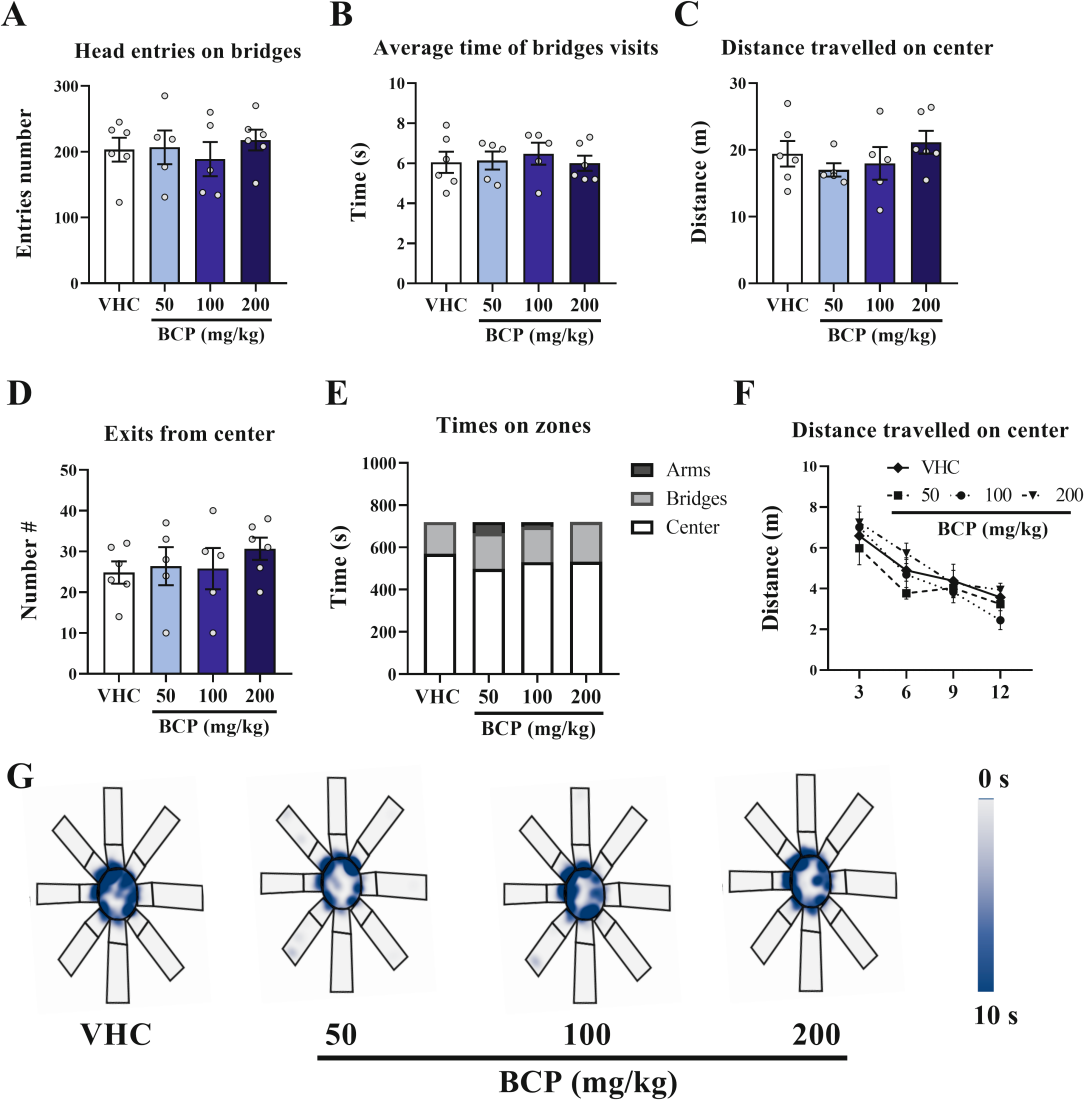
Anxiety‐like behavior results from acute pharmacological assay by OTP: The 3D maze test was performed 30 min after VHC or BCP oral administrations and the test lasted 12 min, as well as the entries schedule were randomly between groups. (A) Number of head entries on bridges. (B) Average time of bridges visits (s). (C) Distance travelled on center (m). (D) Number of exits from center. (E) Time spent on zones (s). (F) Distance travelled on center by segments of test; (G) Heat map of groups' trajectory. All data were expressed as mean (E) or mean ± SEM (A, B, C, D, F), whereas the time in zones were expressed as mean. At all analyses, there are no significant differences between groups: *p* > 0.05.

**FIGURE 3:**
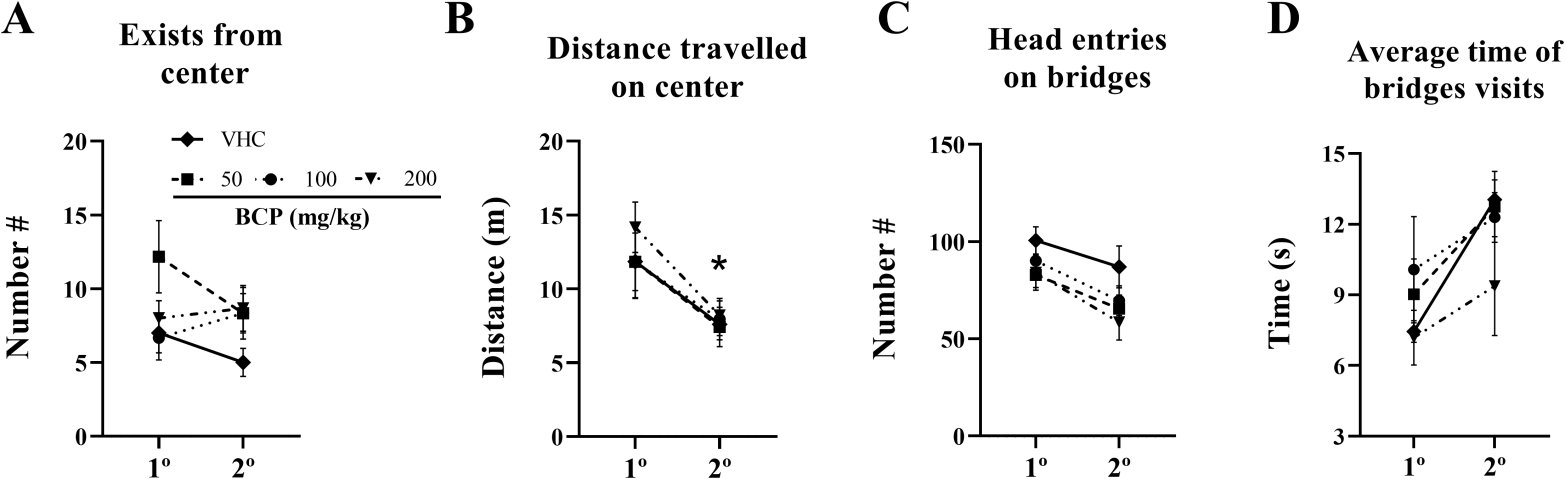
ALEB results from acute pharmacological assay by TTP: The 3D maze test was performed 30 min after VHC or BCP oral administrations and the test lasted 12 min (2 min of interval between 2 trails of 5 min), as well as the entries schedule were randomly between groups. (A) Number of exits from center. (B) Distance travelled on center (m). (C) Number of head entries on bridges. (D) Average time of bridges visits (s). All data were expressed as mean ± SEM At all analyses, there are no significant differences between groups in each trial: *p* > 0.05. *p < 0.05 between trails in all groups.

For OTP or in each trial of TTP, there are no differences between groups over all parameters measured: number of entries with head on the bridges, average time spent on the bridges, distance traveled on the center, number of exits from the center, times on the zones (center, bridges, arms), and distance traveled on the center for 4 segments of 3 min of test: *p* > 0.05 at all parameters (Figure 2; Figure 3).

In addition, we compared the data of OTP (first and second 5 min of 12 min of test) with TTP. The results support that TTP promotes a significant difference between trials of all groups in immobile time (*p* < 0.05 for all groups) and distance traveled in the center (*p* < 0.05 for all groups), whereas in OTP, there is no difference between first and second 5 min over these same parameters (Figure S3).

### 3.3 BCP Chronic‐Treatment Does not Promote Effects on Locomotion or ALEB in Swiss Male Mice

On the 1st, 4th, 7th, 10th, and 16th day of treatment, the animal mass was measured. Over these data, we did not find any significant difference between groups or between cages (Figure S4): *p* > 0.05 in all analyses, which suggests the same environmental conditions for all groups or cages of animals.

**FIGURE 4:**
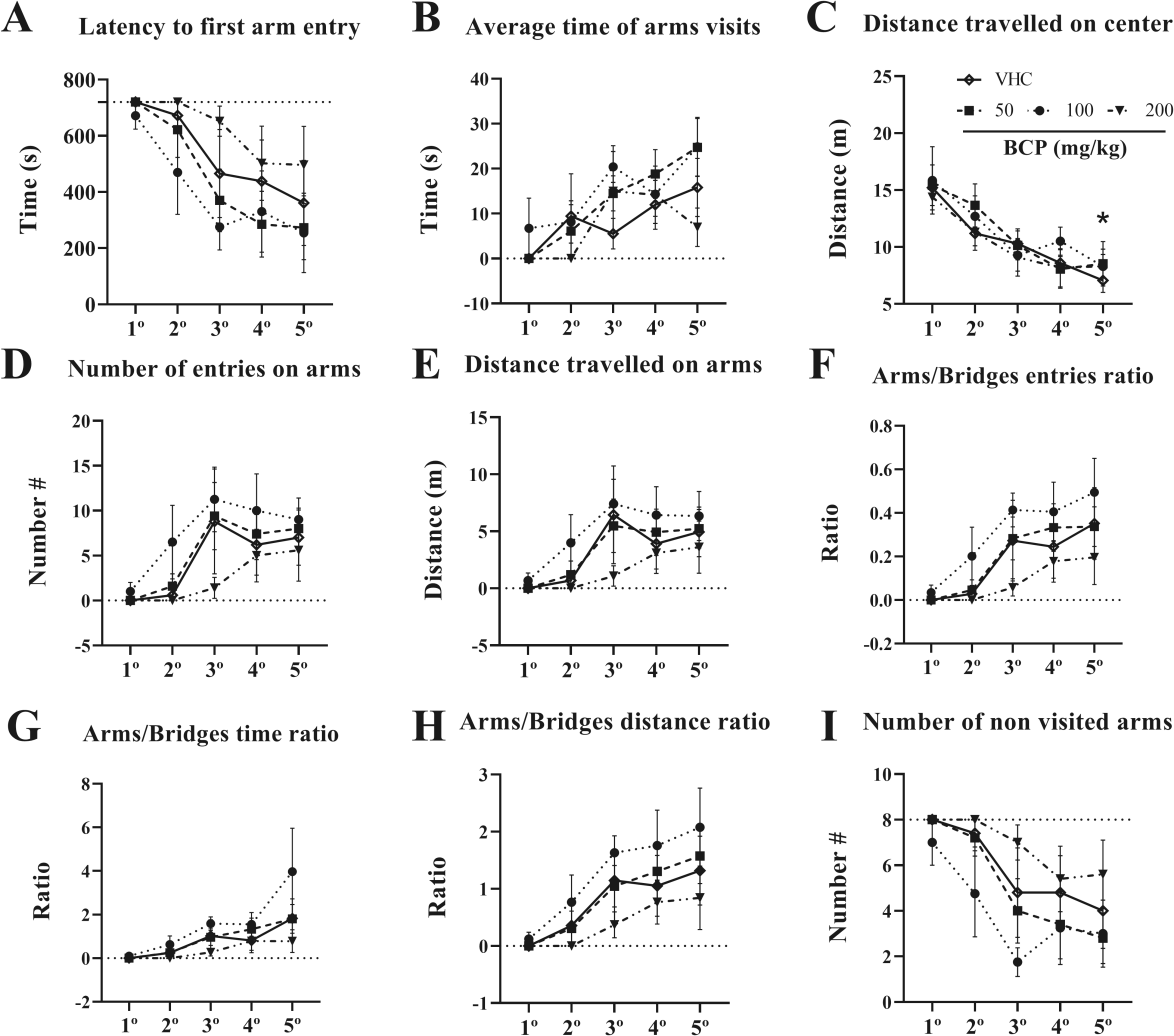
Anxiety‐like behavior results from chronic pharmacological assay: For 5 consecutive days, the 3D maze test was performed 30 min after VHC or BCP oral administrations and the test lasted 12 min, as well as the entries schedule were randomly between groups. (A) Latency to first arm entry (s). (B) Average time of arms visits (s). (C) Distance travelled on center (m). (D) Number of entries on arms. (E) Distance travelled on arms (m). (F) Arms/bridges entries ratio. (G) Arms/bridges time ratio. (H) Arms/bridges distance ratio. (I) Number of non‐visited arms. All data were expressed as mean ± SEM At all analyses, there are no significant differences between groups: *p* > 0.05. * *p* < 0.05 between 1° vs. 5° trial of each group.

Over the 11th to 15th day of treatment, 3D maze tests were performed. Along with the five sessions of tests, we observed a decrease in anxiety‐like behavior across all groups: latency to first arm entry decreased 359 s for vehicle, 447 s for BCP50, 417 s for BCP100, and 223 s for BCP200 between the 1st and 5th session (*p* = 0.1924, *p* = 0.0461, *p* = 0.1122, and *p* > 0.05, respectively). Average time of arms visit increased 15.82 s for vehicle, 24.74 s for BCP50, 18.13 s for BCP100, and 6.96 s for BCP200 between the 1st and 5th session (*p* = 0.1793, *p* = 0.0484, *p* = 0.0724, and *p* > 0.05, respectively). Center distance traveled decreased 8.154 m for vehicle, 6.902 m for BCP50, 7.552 m for BCP100, and 6.074 m for BCP200 between the 1st and 5th session (*p* = 0.0001, *p* = 0.0001, *p* = 0.0002, and *p* = 0.0008, respectively). Number of arm entries increased 7 for vehicle, 8 for BCP50, 8 for BCP100, and 6 for BCP200 between the 1st and 5th session (*p* = 0.211, *p* = 0.659, *p* = 0.1030, and *p* > 0.05, respectively). Arms distance traveled increased 4.945 m for vehicle, 5.229 m for BCP50, 5.669 m for BCP100, and 3.61 m for BCP200 between the 1st and 5th session (*p* = 0.2131, *p* = 0.0952, *p* = 0.1621, and *p* > 0.05, respectively). Arms/bridges entries ratio increased 0.3521 for vehicle, 0.3371 for BCP50, 0.4601 for BCP100, and 0.1965 for BCP200 between the 1st and 5th session (*p* = 0.2437, *p* = 0.0551, *p* = 0.0978, and *p* > 0.05, respectively). Arms/bridges time ratio increased 1.829 for vehicle, 1.805 for BCP50, 3.875 for BCP100, and 0.782 for BCP200 between the 1st and 5th session (*p* = 0.2681, *p* = 0.1348, *p* = 0.3085, and *p* > 0.05, respectively). Arms/bridges distance ratio increased 1.317 for vehicle, 1.574 for BCP50, 1.958 for BCP100, and 0.8422 for BCP200 between 1st and 5th session (*p* = 0.2335, *p* = 0.0817, *p* = 0.1199, and *p* > 0.05, respectively). Number of no visit arms decreased 4 for vehicle, 5 for BCP50, 4 for BCP100, and 2 for BCP200 between the 1st and 5th session (*p* = 0.1811, *p* = 0.0260, *p* = 0.2555, and *p* > 0.05, respectively) (Figure 4).

**FIGURE 5:**
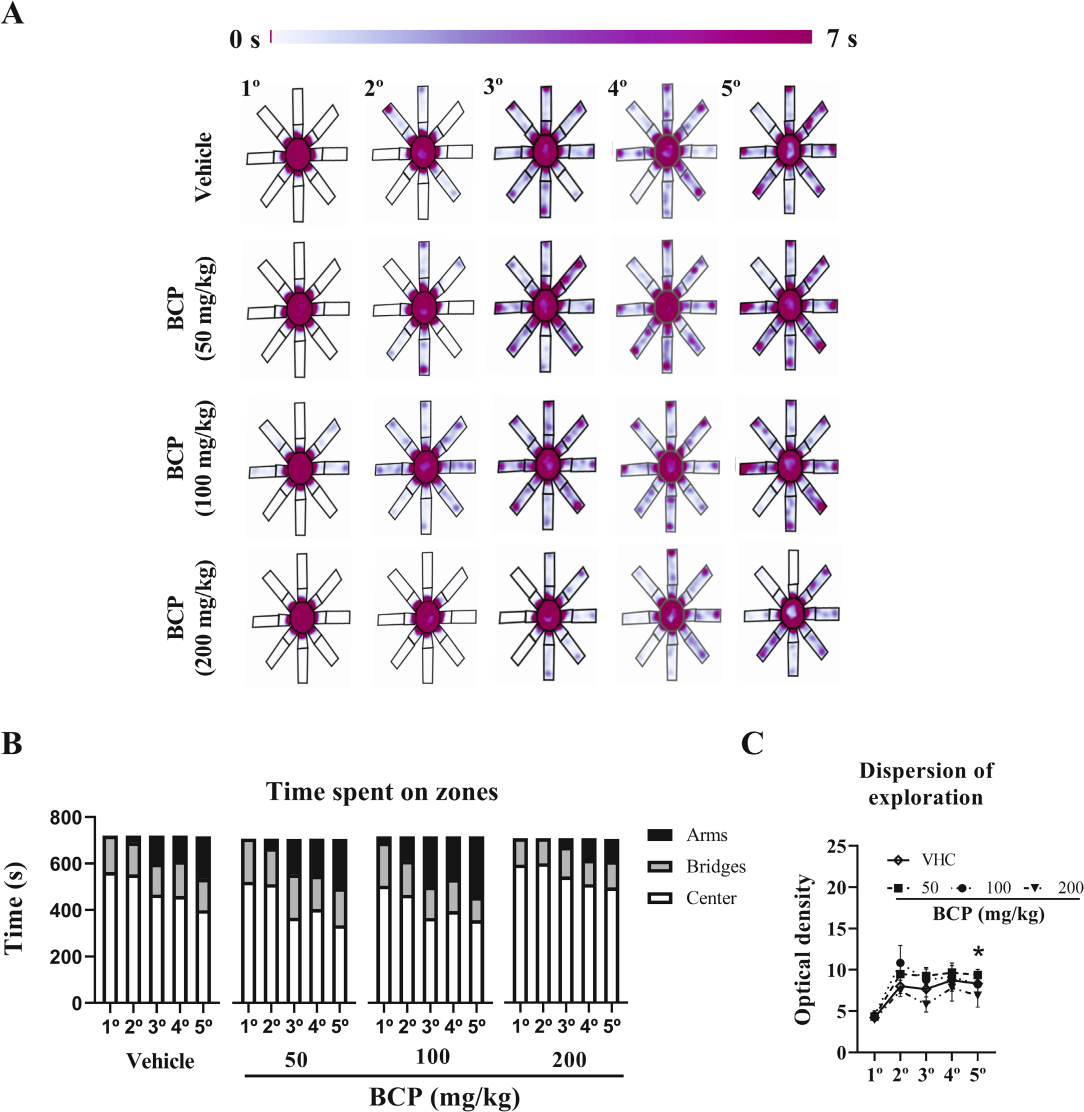
Time spent on zones from chronic pharmacological assay: For 5 consecutive days, the 3D maze test was performed 30 min after VHC or BCP oral administrations and the test lasted 12 min, as well as the entries schedule were randomly between groups: (A) heat map of all group during 5 days of experiment. (B) Time spent on zones (arms, bridges, and center). (C) Dispersion of exploration: for this analysis, the optical density of heat map in grayscale was performed using Fiji ImageJ software (version 1.54, National Institutes of Health, United States). All data were expressed as mean (B) or mean ± SEM (C). At all analyses, there are no significant differences between groups: *p* > 0.05; * *p* < 0.05 between 1° vs. 5° trial of each group.

Furthermore, in overall groups, the time on center decreased (1st vs. 5th: *p* < 0.05 for vehicle, BCP50, and BCP100; *p* > 0.05 for BCP200) whereas time on arms increased (1st vs. 5th: *p* = 0.3234 for vehicle; *p* = 0.1003 for BCP50; *p* = 0.2087 for BCP100; and *p* > 0.05 for BCP200) along to 5 sessions of the 3D maze test (Figure 5B). Likewise, the dispersion of exploration does not show the difference between groups over any day; however, in overall groups, there are significant differences between 1st and 5th day (*p* > 0.05), which raised a higher dispersion by time. For this analysis, the optical density of the heat map in grayscale was performed using Fiji ImageJ software (version 1.54, National Institutes of Health, United States) (Figure 5C).

**FIGURE 6:**
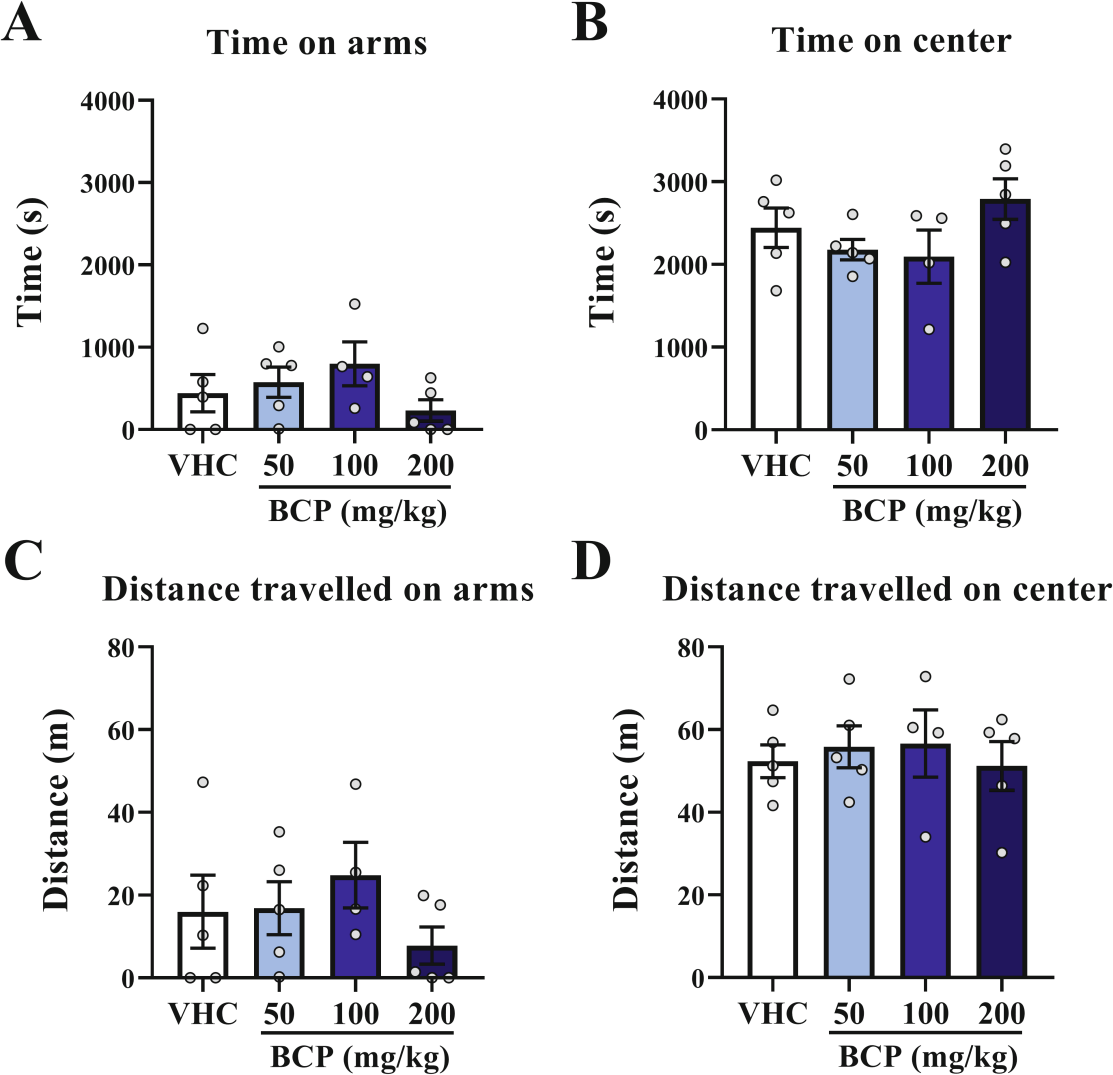
Cumulative parameters of anxiety‐like behavior result from chronic pharmacological assay: For 5 consecutive days, the 3D maze test was performed 30 min after VHC or BCP oral administrations and the test lasted 12 min, as well as the entries schedule were randomly between groups. (A) Time spent on arms (s). (B) Time spent on center (s). (C) Distance travelled on arms (m). (D) Distance travelled on center (m). All data were expressed as mean ± SEM At all analyses, there are no significant differences between groups: *p* > 0.05.

In addition, over all these parameters, there are no significant differences between BCP treatments against the vehicle group (*p* > 0.05 for all analyses), even over cumulative (all 5 sessions of the 3D test) time spent, or distance traveled on center or arms (Figure 6).

**FIGURE 7:**
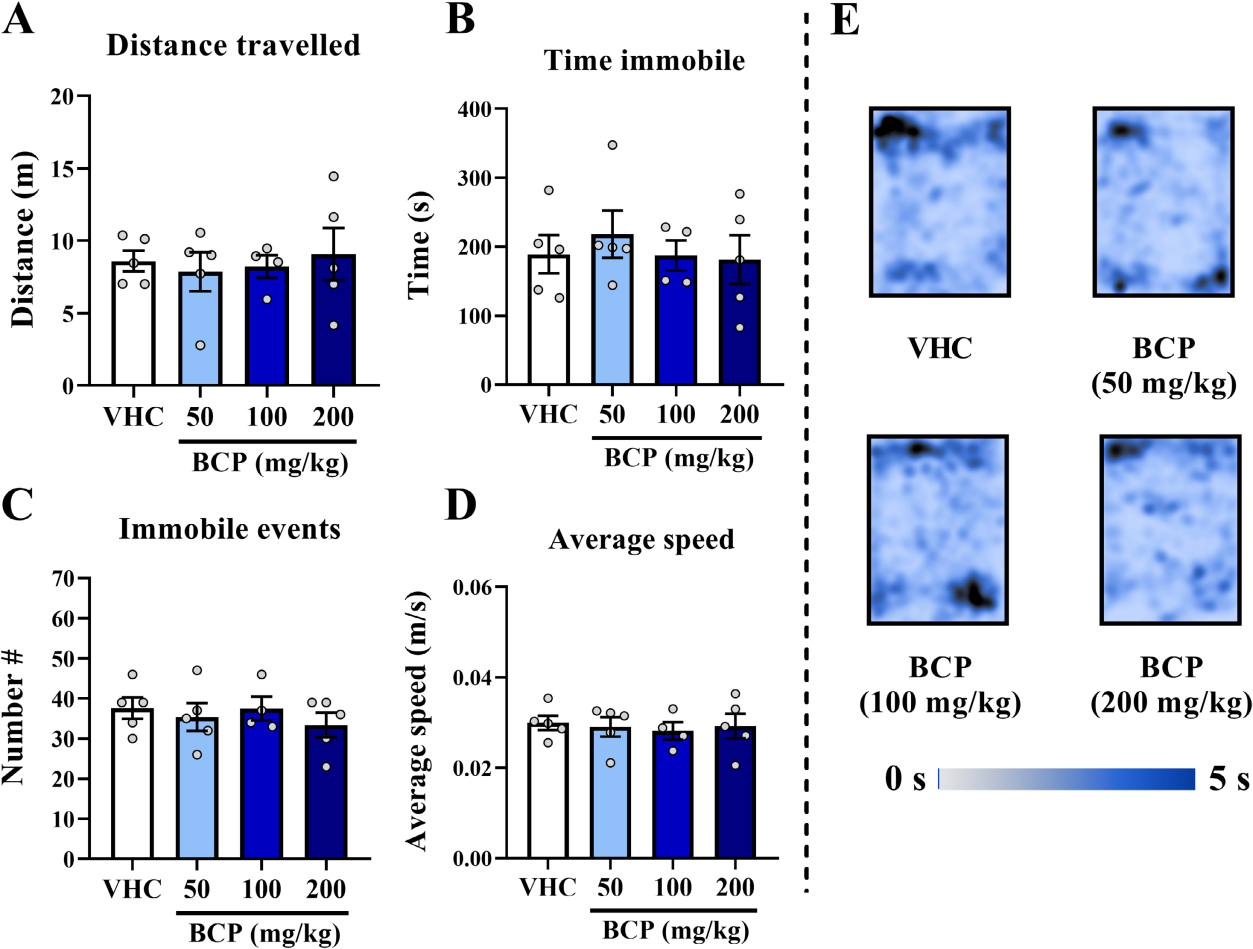
Locomotion results from chronic pharmacological assay: The locomotion test was performed 30 min after VHC or BCP oral administrations and the test lasted 10 min, as well as the entries schedule were randomly between groups. (A) Distance travelled (m). (B) Immobile time (s). (C) Number of immobile events. (D) Average speed (m/s): calculated by distance travelled divided by mobile time. (E) Heat map of groups' trajectory. All data were expressed as mean ± SEM At all analyses, there are no significant differences between groups: *p* > 0.05.

In the last analysis, over the 18th day of treatment, we performed a locomotion test. For distance traveled, immobile time, number of immobile events, or average speed, there are no significant differences between all groups (*p* > 0.05) (Figure 7). As well as over these same parameters analyzed in the 3D maze test, there are also no differences between groups over all five sessions (Figure S5).

## 4 Discussion

The pharmacotherapy for psychological disorders is dependent on safe chronic treatments. In this sense, according to our knowledge, this is the first study that assessed ALEB over BCP chronic treatment. Likewise, acute outcomes must be investigated precisely and over various conditions, especially in anxiety‐like behavior assessment. In addition, abolishing confounded results must be sought; thus, locomotion alterations seem to be the primary target for this issue in mouse models.

In this work, concerning acute or chronic conditions, systemic BCP treatment was not able to promote effects on locomotion in male Swiss mice. Over CB2R knockout conditions, Ortega‐Alvaro et al. (2011) reported a decrease in locomotion in the open‐field test (OFT) in mice [28], whereas Li and Kim (2016) reported no effects on locomotion in OFT in mice [29]. Nevertheless, the endocannabinoid system is present in CNS development [30], which impaired studies with knockout mice for investigations of CB2R function. However, locomotion modulation by CB2R impressions is associated with dopaminergic neurons or dopaminergic metabolism [31, 32].

Acute and systemic BCP administration has not promoted alterations in locomotion in OFT at doses of 50, 100, 200, or 400 mg/kg [16, 17, 18]. However, dependent on CB2R presence, BCP treatment modulates addiction in rats by dopaminergic pathways [33]. Thus, CB2R present in dopaminergic neurons seems to modulate addiction and dopaminergic metabolism [13, 14, 31, 32, 33], which makes the CB2R a pharmacological target for psychological conditions associated with motivation. On the other hand, in this and previous works, BCP systemic administration has not promoted locomotion alterations in mice.

Concerning anxiety‐like behavior, we reported no effects induced by systemic BCP acute treatment over 2 conditions in the 3D maze test: OTP without habituation (high anxiety spectrum) and TTP with habituation (low anxiety spectrum). Differently, systemic BCP acute treatment has been reported as an anxiolytic treatment in current anxiety‐like behavior unconditioned tests, i.e., OFT, Elevated Plus Maze test (EPM), and light–dark box test, in male and female mice [16, 17, 18]. As well as in a pilot test performed in our laboratory, oral administration of 50 mg/kg of BCP promotes a significant increase in the distance traveled in the center of OFT in Swiss mice 2 h after the treatment (data not shown). Current tests for assessing anxiety keep an avoid/escape space (e.g., closed arms, walls, corners, or dark chamber) that provides intrinsic conflict between the preference for a secure place and motivation to explore, as well as these current tests could not be sensitive to subsequent sessions and provide few parameters for analysis, whereas all these flaws are absent in the 3D maze test [20].

On the other hand, our data does not suggest BCP as an anxiety modulator in a 3D maze. In this sense, the overexpression of CB2R in glutamatergic neurons from the hippocampus in mice also promotes an increase in the time spent in the central area of the OFT whereas it had no effect in the zero maze [34], which suggests that this effect is disassociated from anxiety‐like behavior. Therefore, this increase in aversive areas of current anxiety‐like behavior unconditioned tests may be linked to dopaminergic modulation by neuronal CB2R.

In addition, over all anxiety‐like behavior assessing tests performed in this work, the groups express high motor activity in the first minutes or first trials; this hyperactivity could be interpreted as an avoidance behavior induced by low habituation. However, in this work, it should not be disassociated from anxiety. Indeed, over pharmacological trials, hyperlocomotion could be distinguished from anxiety [35]. Moreover, hyperlocomotion is associated with stress in mice [36], as well as over new and anxiogenic conditions (OFT and EPM). Some types of neurons in the basolateral amygdala are activated progressively and stronger along subsequently sections [37], which could be correlated with anxiety‐related or habituation‐related behavioral changes.

Nevertheless, at this work, BCP systemic treatments have not modulated it. Besides, we report decreased motor activity in the center of the 3D maze as an anxiolytic‐like effect induced by habituation or experience; this effect is stronger in TTP against OTP, as well as motor activity was decreased also over subsequent segments OTP (not significantly) and, together with other parameters of anxiety‐like behavior, over five sessions of BCP chronic assay in the 3D maze in all groups. Thus, we suggest that TTP promotes a small window of 12 min (2 trials of 5 min and an interval of 2 min between both), where there is a high anxiogenic context (first trial) and low anxiogenic context (second trial) induced by 2 min of interval. In this way, our data suggest that TTP in the 3D maze could be a robust tool for assessing anxiolytic drug effects at the first trial and anxiogenic drug effects at the second trial in the pharmacological acute assays. This decreased anxiety‐like behavior over time is memory‐dependent in mice [27], which could confound anxiety‐like behavior assessment.

Concerning chronic assay, the three doses of BCP have not promoted effects in anxiety‐like behavior or ALEB assessed by the 5‐day protocol of the 3D maze. Likewise, anxiety‐like behavior decreased over days in all groups analyzed, supported by enhancement of exploration onto arms (anxiolytic effect). Interestingly, the 10‐day chronic treatments have not promoted significantly decreased anxiety‐like behavior (entrances onto arms) in Swiss male mice in contrast to acute treatment as previously reported in Balb‐c mice [21]. We suppose that this issue could be argued by oral and not intraperitoneal administration in this work or by the difference in the strains. However, it remains unclear and must be investigated in the future.

Lastly, the 3D maze test was raised as a tool for assessing chronic pharmacological assays in mouse models, which is essential for searching for safe chronic treatments with drugs for psychological disorders. In contrast, current tests for assessing anxiety in mice are not sensitive to subsequent trials [20], which disturbs chronic pharmacological analyses. In addition, recently, a clinical trial has not found BCP effects on depression, anxiety, or stress using a scale‐21 in obese women diagnosed with food addiction [38]. However, our results were performed in healthy animals, and despite the BCP pharmacological targets not being exclusive to CB2R [39], the CB2R behavior in many models and diseases supports that receptor as a therapeutic target to be explored in psychological conditions [40, 41]. Thus, clinical trials with anxiety patients must be performed to corroborate these results.

## 5 Conclusion

Thus, despite its immunomodulatory effects, BCP systemic treatment, acute or chronic, suggests that it does not promote effects on anxiety‐like behavior, ALEB, or locomotion in healthy Swiss male mice. Furthermore, the presence of CB2Rs in neurons and glial cells as well as in immunological cells disturbs the investigation of its functions. However, BCP is raised as a promising drug for inflammatory diseases; nonetheless, the effects on CNS must be researched to unravel possible treatments for other neurological conditions such as psychological diseases or for possible side effects.

## Author Contributions

**Rayan Fidel Martins Monteiro**: conceptualization, formal analysis, investigation, methodology, project administration, visualization and writing – original draft preparation. **Marcos Vinícius Lebrego Nascimento**: investigation. **Klinsmann Thiago Lima**: investigation and methodology. **Valdina Solimar Lopes Cardoso**: methodology. **José Ramon Gama Almeida**: investigation. **Wellington Junior Taisho Nagahama Costa**: investigation. **Anderson Valente‐Amaral**: investigation. **Ludmila Santos Barbosa**: investigation. **Vinicius Teles Shirakura**: investigation. **Gilmara de Nazareth Tavares Bastos**: conceptualization, methodology, resources, supervision, writing – review and editing.

## Conflicts of Interest

The authors declare no conflicts of interest.

## Supporting information


**FIGURE S1:** Locomotion results from acute pharmacological assay by segments of the test: the locomotion test was performed 20 min after VHC or BCP oral administrations and the test lasted 20 min, as well as the entries schedule were randomly between groups. (A) Distance travelled (m). (B) Immobile time (s). All data were expressed as mean ± SEM At all analyses, there are no significant differences between groups: *p* > 0.05.
**FIGURE S2:** Locomotion results in 3D maze from acute pharmacological assay: the 3D maze test was performed 30 min after VHC or BCP oral administrations and the test lasted 12 min, as well as the entries schedule were randomly between groups. (A) Distance travelled (m). (B) Immobile time (s). (C) Number of immobile events. (D) Average speed (m/s): calculated by distance travelled divided by mobile time. (E) Distance travelled by segments of the test. (F) Time immobile by segments of the test. All data were expressed as mean ± SEM. At all analyses, there are no significant differences between groups: p > 0.05.
**FIGURE S3:** Anxiety‐like behavior results from acute pharmacological assay by OTP and TTP: the 3D maze test was performed 30 min after VHC or BCP oral administrations and the test lasted 12 min (2 min of interval between 2 trails of 5 min) for TTP and 12 min for OTP, as well as the entries schedule were randomly between groups at both protocols. (A) TTP: distance travelled on center (m). (B) OTP: distance travelled on center (m). (C) TTP: immobile time (s). (D) OTP: immobile time (s). All data were expressed as mean ± SEM. At all analyses, there are no significant differences between groups in each trial: p > 0.05. *p < 0.05 between trails in all groups.
**FIGURE S4:** Body mass. The body mass was recorded in the 1st, 4th, 7th, 10th, and 16th day of chronic pharmacological assay. (A) Body mass between groups. (B) Body mass between cages. All data were expressed as mean ± SEM. At all analyses, there are no significant differences between groups or between cages in each day: p > 0.05.
**FIGURE S5:** Locomotion results in 3D maze from chronic pharmacological assay: for 5 consecutive days, the 3D maze test was performed 30 min after VHC or BCP oral administrations and the test lasted 12 min, as well as the entries schedule were randomly between groups. (A) Distance travelled (m). (B) Immobile time (s). (C) Number of immobile events. (D) Average speed (m/s): calculated by distance travelled divided by mobile time. All data were expressed as mean ± SEM. At all analyses, there are no significant differences between groups: p > 0.05.

## Data Availability

Research data are not shared.
