# Supplementary material for: Acute or Chronic β‐Caryophyllene Systemic Administration in Healthy Adult Male Mice Does Not Modulate Anxiety‐Like Extinction Behavior Induced by Subsequent Re‐Exposure to 3D Maze
Source: Fundam Clin Pharmacol. 2025 Sep 26;39(6):e70049. doi: 10.1111/fcp.70049 (PMC12466847; doi:10.1111/fcp.70049)
Supplement: Supplementary file 1 — FIGURE S1: Locomotion results from acute pharmacological assay by segments of the test: the locomotion test was performed 20 min after VHC or BCP oral administrations and the test lasted 20 min, as well as the entries schedule were randomly between groups. (A) Distance travelled (m). (B) Immobile time (s). All data were expressed as mean ± SEM At all analyses, there are no significant differences between groups: p > 0.05. FIGURE S2: Locomotion results in 3D maze from acute pharmacological assay: the 3D maze test was performed 30 min after VHC or BCP oral administrations and the test lasted 12 min, as well as the entries schedule were randomly between groups. (A) Distance travelled (m). (B) Immobile time (s). (C) Number of immobile events. (D) Average speed (m/s): calculated by distance travelled divided by mobile time. (E) Distance travelled by segments of the test. (F) Time immobile by segments of the test. All data were expressed as mean ± SEM. At all analyses, there are no significant differences between groups: p > 0.05. FIGURE S3: Anxiety‐like behavior results from acute pharmacological assay by OTP and TTP: the 3D maze test was performed 30 min after VHC or BCP oral administrations and the test lasted 12 min (2 min of interval between 2 trails of 5 min) for TTP and 12 min for OTP, as well as the entries schedule were randomly between groups at both protocols. (A) TTP: distance travelled on center (m). (B) OTP: distance travelled on center (m). (C) TTP: immobile time (s). (D) OTP: immobile time (s). All data were expressed as mean ± SEM. At all analyses, there are no significant differences between groups in each trial: p > 0.05. *p < 0.05 between trails in all groups. FIGURE S4: Body mass. The body mass was recorded in the 1st, 4th, 7th, 10th, and 16th day of chronic pharmacological assay. (A) Body mass between groups. (B) Body mass between cages. All data were expressed as mean ± SEM. At all analyses, there are no significant differences between groups or bet [file FCP-39-0-s001.docx]

**Supporting information**


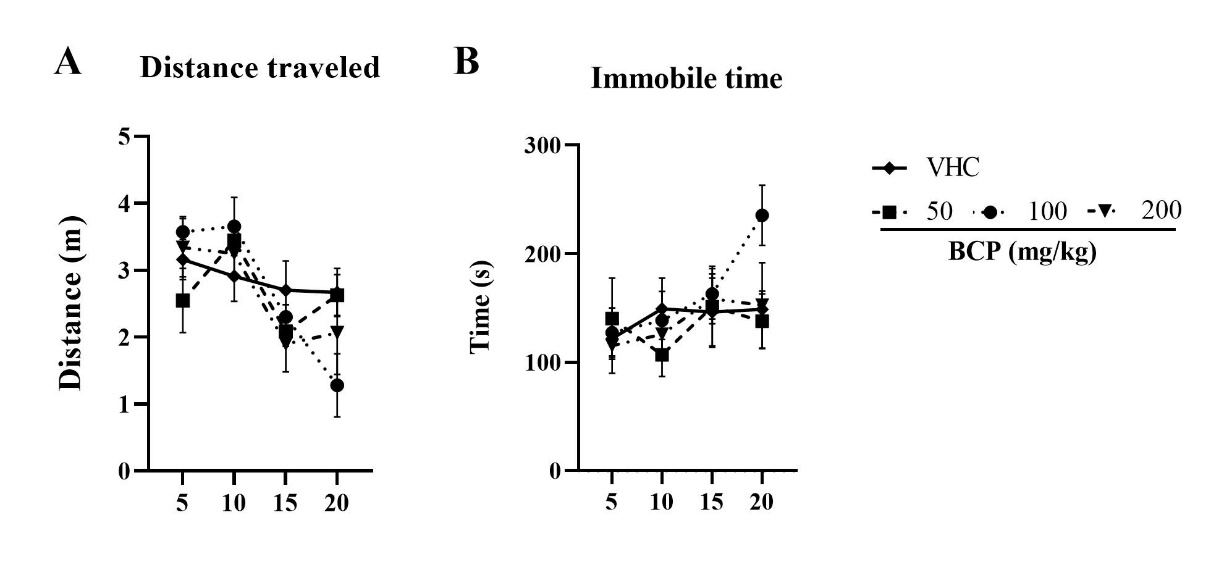


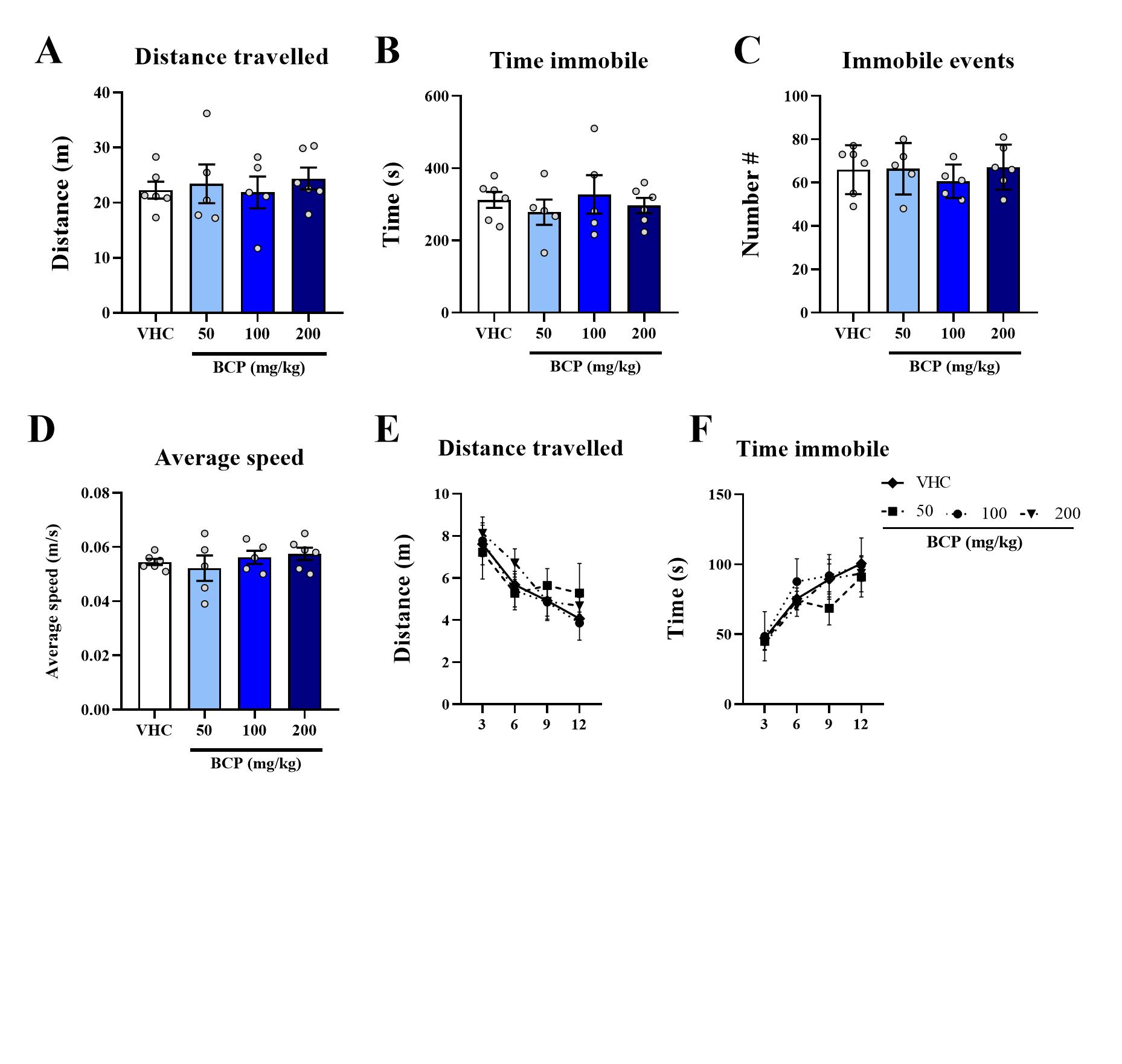
**Figure S1**. **Locomotion results from acute pharmacological assay by segments of the test**: The locomotion test was performed 20 min after VHC or BCP oral administrations and the test lasted 20 min, as well as the entries schedule were randomly between groups. (**A**) Distance travelled (m); (**B**) Immobile time (s). All data were expressed as mean ± SEM. At all analyses there are not significant differences between groups: *p* > 0,05.

**Figure S2**. **Locomotion results in 3D maze from acute pharmacological assay**: The 3D maze test was performed 30 min after VHC or BCP oral administrations and the test lasted 12 min, as well as the entries schedule were randomly between groups. (**A**) Distance travelled (m); (**B**) Immobile time (s); (**C**) Number of immobile events; (**D**) Average speed (m/s): calculated by distance travelled divided by mobile time; (**E**) Distance travelled by segments of the test; (**F**) Time immobile by segments of the test. All data were expressed as mean ± SEM. At all analyses there are not significant differences between groups: *p* > 0,05.


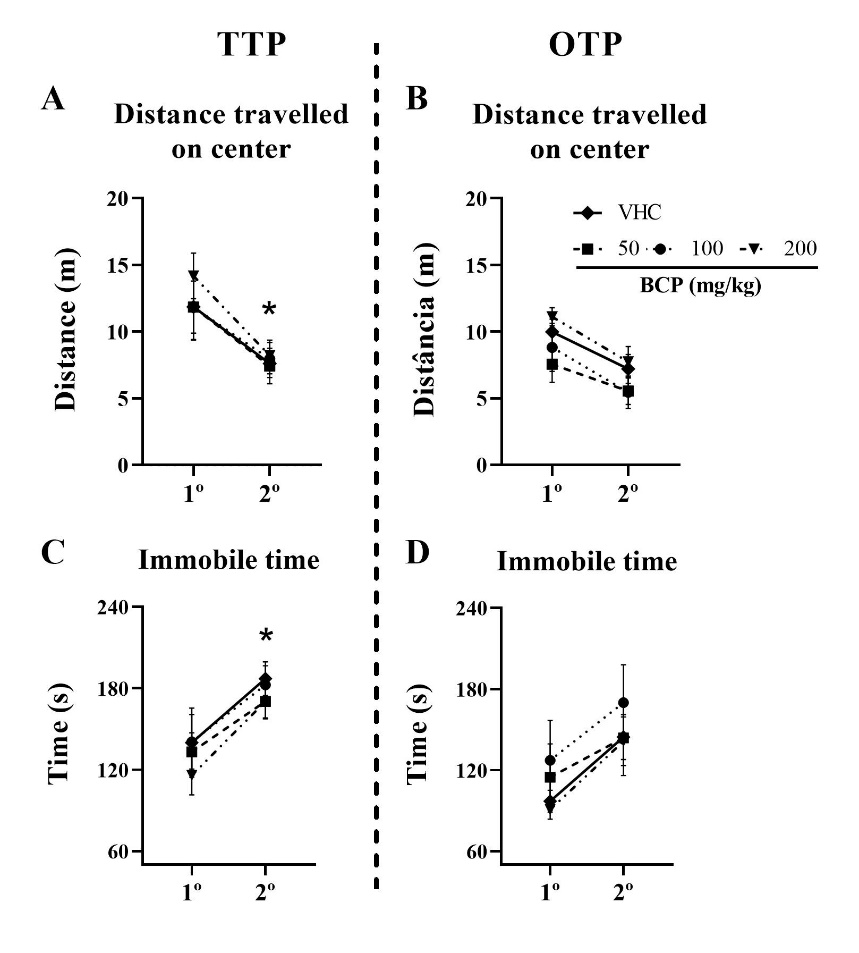


**Figure S3**. **Anxiety-like behavior results from acute pharmacological assay by OTP and TTP**: The 3D maze test was performed 30 min after VHC or BCP oral administrations and the test lasted 12 min (2 min of interval between 2 trails of 5 min) for TTP and 12 min for OTP, as well as the entries schedule were randomly between groups at both protocols. (**A**) TTP: Distance travelled on center (m); (**B**) OTP: Distance travelled on center (m) (**C**) TTP: Immobile time (s); (**D**) OTP: Immobile time (s). All data were expressed as mean ± SEM. At all analyses there are not significant differences between groups in each trial: *p* > 0,05. **p* < 0,05 between trails in all groups.


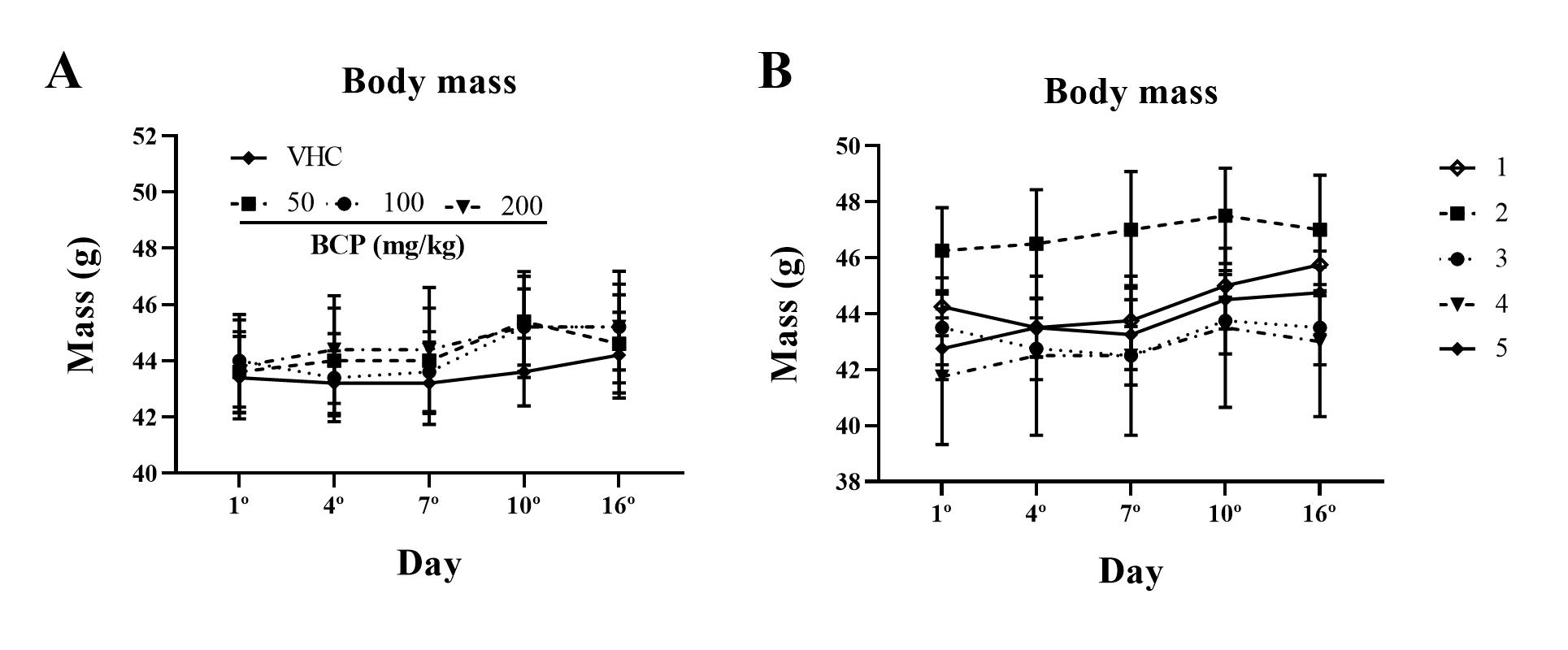


**Figure S4**. **Body mass**. The body mass was recorded in the 1^st^, 4^th^, 7^th^, 10^th^ and in 16^th^ day of chronic pharmacological assay. (**A**) Body mass between groups; (**B**) Body mass between cages. All data were expressed as mean ± SEM. At all analyses there are not significant differences between groups or between cages in each day: *p* > 0,05.

**
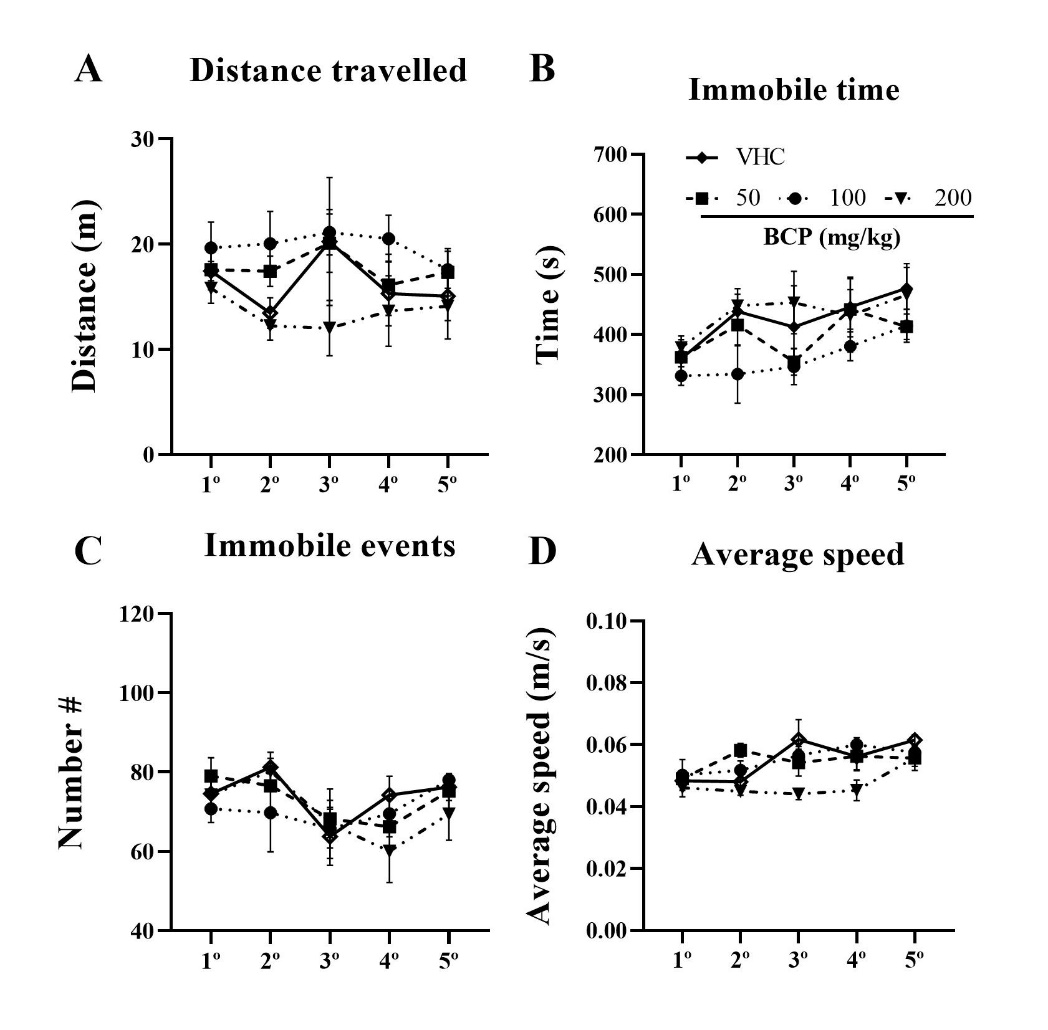
Figure S5**. **Locomotion results in 3D maze from chronic pharmacological assay**: For 5 consecutive days the 3D maze test was performed 30 min after VHC or BCP oral administrations and the test lasted 12 min, as well as the entries schedule were randomly between groups: (**A**) Distance travelled (m); (**B**) Immobile time (s); (**C**) Number of immobile events; (**D**) Average speed (m/s): calculated by distance travelled divided by mobile time. All data were expressed as mean ± SEM. At all analyses there are not significant differences between groups: *p* > 0,05.
